# Systematic review of the changes in the microbiome following spinal cord injury: animal and human evidence

**DOI:** 10.1038/s41393-021-00737-y

**Published:** 2022-01-06

**Authors:** Ezra Valido, Alessandro Bertolo, Gion Philip Fränkl, Oche Adam Itodo, Tainá Pinheiro, Jürgen Pannek, Doris Kopp-Heim, Marija Glisic, Jivko Stoyanov

**Affiliations:** 1 Swiss Paraplegic Research, Nottwil, Switzerland; 2 Department of Health Sciences, University of Lucerne, Lucerne, Switzerland; 3 Department of Orthopedic Surgery, University of Bern, Bern Inselspital, Bern, Switzerland; 4 Graduate School of Cellular and Biomedical Science, University of Bern, Bern, Switzerland; 5 Institute of Social and Preventive Medicine, University of Bern, Bern, Switzerland; 6 Neuro-Urology, Swiss Paraplegic Center, Nottwil, Switzerland; 7 Department of Urology, Inselspital, Bern University Hospital, University of Bern, Bern, Switzerland; 8 Public Health & Primary Care Library, University Library of Bern, University of Bern, Bern, Switzerland

**Keywords:** Infection, Bacterial infection

## Abstract

**Study design:**

Systematic review.

**Objectives:**

To investigate the changes in the microbiome among human and animal populations with spinal cord injury (SCI).

**Methods:**

Four databases (EMBASE, Medline (Ovid), Web of Science, Cochrane Central Register of Trials (CENTRAL)) and Google Scholar were searched. No language restrictions were applied. Data extraction was done in parallel and independently by two reviewers. The search was last conducted on 07 April 2021.

**Results:**

There were 6869 studies retrieved, 43 full-text studies reviewed, and 19 studies included. There were seven animal gut studies, six human gut studies, and six urinary tract studies identified. There were no publications found on other body sites. Among the included studies, we observed a consistent and significant difference in gut microbiome composition between populations with SCI and able-bodied populations. This is characterized by a decrease in beneficial butyrate-producing bacteria (*Faecalbacterium*, *Megamonas*, *Roseburia*) and an increase in inflammation-associated bacteria (*Alistipes*, *Anaerotruncus*, and *Lachnoclostridium*). On the other hand, the urine of individuals with SCI was polymicrobial and members of Enterobacteriaceae (*Escherichia coli*, *Klebsiella pneumoniae*) were frequently observed. Probiotics were shown to induce a significant but transient shift in the urinary tract microbiome. The studies had low to moderate risks of bias.

**Conclusions:**

There are limited studies on the changes in microbiome among SCI populations. The gut microbiome was characterized by bacterial profiles associated with chronic inflammation and metabolic disorder while the studies of the urinary tract microbiome show the dominance of bacterial genera associated with urinary tract infection.

## Introduction

After spinal cord injury (SCI), the loss of innervation on organ systems below the level of lesion induces changes that affect basic body functions. This disturbs the gut and urinary tract motility, glandular secretions, and the overall tone of vascular vessels, muscles, and skin. These changes influence the availability and distribution of nutrients and metabolites needed for the growth of indigenous microbial communities. Depletion or enrichment of microorganisms at an organ-system level is called dysbiosis and can lead to higher risks of infections and increased risks of chronic illnesses such as metabolic disorders, cardiovascular-related morbidities, and autoimmune diseases [1]. Thus, investigating the changes in the microbial communities can impact clinical decisions for individuals with SCI.

Traditionally, the investigation of dysbiosis is through microflora studies via microbial culture methods. The developments in genome sequencing allowed better microbial characterization and identification by studying microbiomes or the collective genome of microorganisms in a specific organ or location at a certain time point [2]. This technique identified previously undetected species and provided evidence of microbial presence on body sites thought to be sterile. Microbiome studies have increased steadily but remained focused on able-bodied individuals with diseases. Growing evidence of specific organisms associated with illnesses is changing medical therapy and therefore understanding the changes in the microbiomes of individuals with SCI can improve medical therapies in this population.

There are narrative reviews regarding gut microbiomes among individuals with SCI [1, 3, 4]. The focus of these reviews is on the inter-relationship of the physiological changes after injury, the immune response, and how the microbiome has changed. These reviews are not systematic and did not explore systematically the bacterial taxa that are enriched or depleted in the microbiome of individuals with SCI. Moreover, to our knowledge, there are no reviews summarizing the evidence on the other body locations in this population. Thus, this systemic review aims to provide a comprehensive overview of the literature concerning the changes in the microbiome in humans and animals with SCI, identify literature gaps and critically appraise the quality of the existing evidence to provide directions for future research.

## Methods

### Data sources and search strategy

This review was conducted in accordance with Preferred Reporting Items for Systematic Reviews and Meta-Analyses [5] and the steps described by Muka et al. [6]. The search strategy was created by an experienced information specialist using terms related to SCI such as paraplegia, tetraplegia, and nontraumatic causes such as spina bifida. These terms were combined with terms for microbiome, microbiota and terms related to their changes such as dysbiosis. Details of the search strategy are provided in Supplementary A. Four electronic databases were searched (EMBASE, Medline (Ovid), Web of Science, the Cochrane Central Register of Controlled Trials (CENTRAL)) and Google Scholar from inception to 07 April 2021 without language restrictions. Detailed study protocol can be found in PROSPERO (CRD42020185555).

### Study selection and eligibility criteria

Studies were included with the following criteria: (1) animal, observational and clinical studies (2) reported the microbial community characteristics through the microbiome via genome or 16S ribosomal ribonucleic acid (16S rRNA) gene sequencing (3) included a population of persons or animals with SCI. Excluded were conference abstracts, reviews, letter to editors, case studies, and non-peer-reviewed studies. Two reviewers independently screened the titles and abstracts for inclusion and assessed eligibility in their full text. Discrepancies were resolved by consensus and if necessary, a third reviewer was consulted.

### Data extraction

Two reviewers independently extracted data using a predesigned table that includes the primary author and year, study characteristics, sequencing technique, microbiome characteristics, diversity indices reported, and other relevant findings. Diversity indices included α and β diversity indices of the microbiome. The α diversity (Chao1, Species or Operational Taxonomic Unit [OTU] or Amplicon Sequence Variant [ASV] counts, ACE, Shannon, Simpson, Inverse Simpson, phylogenetic diversity and Fischer α) measures the diversity within a group while the β diversity (Bray–Curtis, weighted/unweighted UniFrac) measures the diversity between groups [7]. The Human Microbiome Project consortium defines diversity as the number and abundance distribution of specific organisms [8]. The depletion or enrichment of the members of the microbiome were identified as well.

### Assessing the risk of bias

The risk assessment tool used was the Office of Health Assessment & Translation (OHAT) risk of bias tool by the US Department of Health and Human Services [9]. Risk assessment was based on study type. Classification was set with questions on selection, confounding, performance, attrition, and detection biases. Low-risk studies had no probable or definitely high risk or no insufficient information. Moderate-risk studies had probable high risks or insufficient information in any of the biases but without definitely high risk. High-risk studies had a definitely high-risk assessment from one of the biases. Two reviewers independently assessed each study and rated the studies according to OHAT protocol. Discrepancies in risk assessment were resolved by consensus and if needed, consultation with a third reviewer.

## Results

### Characteristics of included studies

**Fig. 1:**
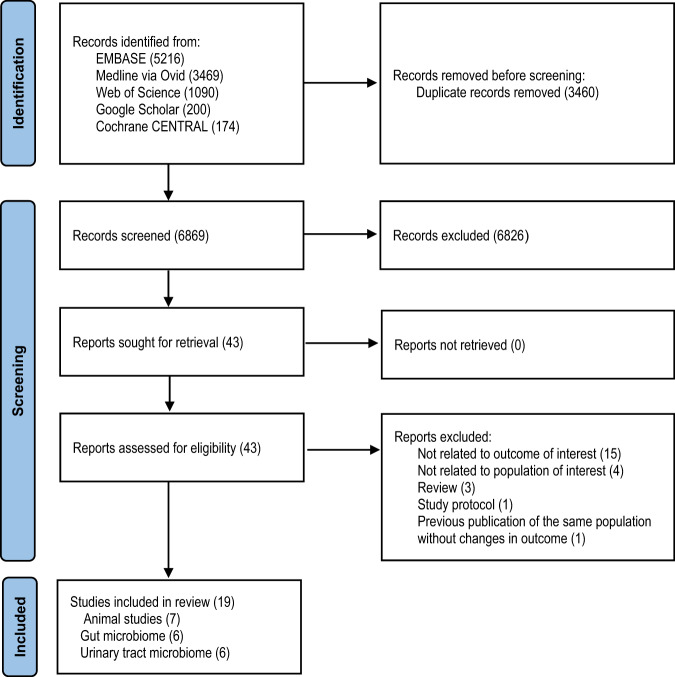
Flowchart of study inclusion. PRISMA flow diagram for the changes in the microbiome following spinal cord injury resulting from searches of databases, screening, reasons for exclusion and description of included studies.

There were 10,329 studies identified. From these, 6869 unique titles and abstracts were screened, 43 full-text studies reviewed of which 19 studies met the inclusion criteria (Fig. 1). Thirteen studies reported on the gut microbiome and six on the urinary tract microbiome. Seven of the studies were animal studies, 12 were human studies with four with interventions and the remaining nine were observational studies. No studies were found on other organ-systems. All studies used the hypervariable regions of bacterial 16S rRNA as sequencing target.

### The gastrointestinal microbiome in animal studies of spinal cord injury

We identified seven rodent SCI studies, four were conducted in mice and three in rats. Five studies included only females. Five studies induced SCI at thoracic (T9/T10) and two at cervical (C5) level. Six studies had intervention in their setting. Two of the studies had a pre-injury baseline of the gut microbial composition. Summarized findings of the microbiome changes can be found in Table 1. Five of the seven animal studies had a low risk of bias. Two had moderate risk and used antibiotics post-injury that could have affected the detected microbiomes [10, 11] (Supplementary Table 1).

**Table 1:** Summary of the findings from the animal gut studies.

Study population	Intervention and experimental arms (*N*)	Sequencing technique	Microbial differences (vs sham injured or non-SCI)	Diversity indices (vs Sham)	Other significant results	RoB assessment (Author, Publication Year, Country)
Female rats w/ laminectomy at T10	Intervention: none Experimental arms: sham injured (16), SCI (16)	16S rRNA V3 + V4 Primers unspecified Illumina MiSeq	↑(f) Clostridiaceae ↑(s) *Lactobacillus intestinalis, Clostridium disporicum, Bifidobacterium choerinum* ↓ (f) Bifidobacteriaceae ↓(s) *Clostridum saccharogumia*	α diversity (−) Shannon β diversity –Difference was found at family, genus, and species by UniFrac-PCoA	IL-1β negatively correlated with *Streptococcus acidominimus*, *Clostridium sp40, Ruminococcus bromii, Faecalibacterium prausnitzii, Gemmiger formicillis, Ruminococcus obeum, Dorea longicatena, Coynebacterium mastitidis* IL 12 negatively correlated with *Lactobacillus intestinalis* and *Bifidobacterium choerinum*. IL 12 positively correlated with *Clostridium saccharogranumia*	Moderate (OConnor et al., 2018 [10], United States)
Adult mice (C57BL/6) with laminectomy at T9	Intervention: probiotic VSL#3, antibiotic cocktail Experimental arms: sham injured, SCI	16S rRNA V4-V5 515F/806R Illumina MiSeq Silva_119	↓(o)Bacteroidales ↑(o)Clostridiales Significant change (o)Anaeroplasmantales, Turicibacterales and Lactobacillales	–	(+) bacterial translocation ↑20% gut permeability in SCI mice ↑ gene expression for proteins for tight junctions and intestinal epithelium differentiation (+) GALT inflammation Antibiotic-induced dysbiosis increases intraspinal inflammation and lowers locomotor recovery	Low (Kigerl et al., 2016 [16], United States)
Female mice with laminectomy at T9	Intervention: Pde4B^−/−^ Experimental arms: sham, SCI, sham injured-SCI w/ Pde4B^−/−^, SCI w/ Pde4B^−/−^	16S rRNA V4 Primers unspecified Illumina MiSeq Greengenes database	↑(p) Bacteroidetes, Proteobacteria ↓(p) Firmicutes		Pde4B^−/−^ ↓chronic inflammation ↑functional recovery – no expansion of Proteobacteria and significant changes in Firmicutes and Bacteroidetes There is 2.5× increase in 16S rRNA gene copy observed after 1 week and sustained for 6 weeks in SCI mice	Low(Myers et al., 2019 [15], United States)
Female mice (C57BL/6) with laminectomy at T10	Intervention: melatonin Experimental arms: sham injured (16), sham injured +melatonin(16), SCI (16), SCI +melatonin (16)	16S rRNA V3 + V4 338F/806R Illumina MiSeq Silva (SSU128) database	↑(o) Clostridiales, ↑(f) Lachnospiracea ↓(o) Lactobacillales, Bifidobacteriales ↓(g) *Lactobacillus*	α diversity Shannon ↑SCI ↑SCI vs SCI + melatonin ACE ↑SCI ↑SCI vs SCI + melatonin β diversity –SCI microbiome clustered distinctly from the sham-operated group by Bray–Curtis-PCoA and PCA	Melatonin ↑gut permeability ↑ gut transit time ↓pro-inflammatory cytokines ↑weight gain Antibiotic introduction lowers locomotor recovery	Moderate (Jing et al., 2019 [12], China)
Female rats with laminectomy at C5	Intervention: FMT Experimental arms: sham injured (11), no injury (10), SCI w/ FMT (14), SCI (10)	16S rRNA V4 Primers unspecified Illumina MiSeq	–	α diversity (−) Shannon β diversity –Proximity of genus-species composition of non-injured mice with SCI w/ fecal transplant by Bray–Curtis- nonmetric multidimensional scaling	FMT ↓gut dysbiosis ↓anxiety like behavior	Low (Schimdt et al., 2020 [13], Canada)
Female rats with laminectomy at C5	Intervention: FMT from anxious donors Experimental arms: SCI + vehicle (15), SCI w/ FMT (15), FMT donors (10)	16S rRNA V3-V4 341F/805R Illumina MiSeq	↑(p) Cyanobacteria, Proteobacteria ↓(g) *Lactobacillus*	α diversity (−) Shannon β diversity –no difference between SCI with FMT or none at genus level	FMT from anxious donors ↑ anxiety like behavior ↑ increased gut permeability	Low (Schmidt et al 2021 [14], Canada)
Female mice (C57BL/6) with laminectomy at T10	Intervention: FMT Experimental arms: sham injured (30), sham injured +FMT (30), SCI (30), SCI + FMT (30)	16S rRNA V3 + V4 338 F/806 R Illumina MiSeq Silva (SSU128) database	↑(p) Bacteroidetes, Proteobacteria ↓(p) Firmicutes ↓(g) *Blautia, Anaerostipes, Lachnospiraceae NK4A136, Christensenellaceae, Lactobacillus*	α diversity ACE ↑SCI Chao ↑SCI β diversity –Difference was found at phylum, genus and ASV by Bray–Curtis-PCoA	FMT ↑ locomotor recovery ↑ restoration of descending motor pathways ↑ neuronal survival and synaptic regeneration ↑ weight gain and metabolic profile ↑ gut transit ↓ neuroinflammation and gut inflammation –maintains instetinal barrier integrity –restores fecal SCFAs –modulates gut microbiome in SCI by ↑ (g) *Blautia, Anaerostipes, Lachnospiraceae NK4A136, Christensenellaceae, Butyrimonas* ↓ (g) *Bilophila*	Moderate (Jing et al., 2021 [11], China)

*RoB* risk of bias, *SCI* spinal cord injury, *RNA* ribonucleic acid, *GALT* gut-associated lymphoid tissue, *IL* interleukin, *PCA* principal component analysis, *PCoA* principal coordinate analysis, *FMT* fecal matter transplant, *SCFA* short-chain fatty acid, *ASV* amplicon sequence variants, *F* forward, *R* reverse, *s* species, *g* genus, *f* family, *o* order, *p* phylum.

The animal studies showed changes in microbial composition after SCI but not necessarily in α diversity. O’Connor et al. [10] compared groups of female rats injured at T10 vs sham-operated rats with the use of gentamicin post-operation and found no significant difference in the Shannon diversity index. There was however significant difference in β diversity at family, genus, and species levels between groups. Jing et al. [11] on the other hand, studied adult female mice with T10 injury vs sham injured mice and compared them to groups with fecal matter transplant (FMT) (SCI-FMT vs sham-FMT). There were significant changes in ACE and Chao diversity indices between SCI mice vs sham injured mice but no difference between SCI-FMT vs the sham group. *Blautia, Anaerostipes, Lachnospiraceae NK4A136, Christensenellaceae* were seen to significantly decrease in the SCI group but were alleviated by FMT. They conclude that FMT significantly influences the microbial community of the gut post-SCI [11]. Jing et al. [12] likewise investigated the use of melatonin (10 mg/kg, twice daily) in female mice with injury at T10. Melatonin led to significantly decreased SCI-induced gut permeability and a decrease in microbial diversity and richness in SCI mice. Schmidt et al. [13] studied a group of female rats with cervical level SCI. There were major differences in composition between the groups of SCI rats vs non-SCI rats and SCI rats vs SCI-FMT rats but not in SCI-FMT rats vs non-SCI rats 3 days post-SCI. The SCI-FMT group had reduced total numbers of altered genus-species OTU suggesting prevention of SCI-induced dysbiosis. By 4 weeks post-SCI, the genus-species OTU difference was reduced compared to pre-injury baseline indicating normalization of the microbiota composition. This is supported by the Shannon diversity index with increased in all groups 3 days post-SCI but were all similar after 4 weeks post-SCI when compared to pre-injury β diversity at the genus-species level indicates proximity in the microbial composition of the groups of SCI-FMT rat and non-SCI group indicating similarity. Schmidt et al. [14] subsequently investigated with FMT from anxious rat donors and confirmed previous data on SCI-induced dysbiosis but there was no difference in β diversity and reduced amount of *Lactobacillus* between SCI rats vs SCI-FMT. This indicates that successful FMT depends on the donors’ microbiome as well [14]. In another study, Myers et al. [15] studied the influence of gram-negative derived lipopolysaccharides on inflammation by provoking endotoxin-mediated cyclic adenosine monophosphate specific Pde4 subfamily b (Pde4b) enzyme induced inflammation in gut dysbiosis post-SCI in female wild type (WT) mice and to mice without the enzyme. The authors reported a 2.5-fold increase in 16 S rRNA gene copy in SCI WT mice compared to sham injured WT mice indicating bacterial enrichment post-SCI. The peak expression of inflammatory markers preceded the significant rise of systemic endotoxemia. Kigerl et al. [16] investigated whether SCI can cause bacterial translocation, the passage of viable bacteria from the gastrointestinal tract to extra-intestinal sites, and changes in gut permeability in mice. They confirmed the bacterial translocation by positive microbial culture of the liver, spleen, kidneys, mesentery, and blood in injured mice and none were found in non-SCI mice after 7 days. This was supported by a 20% increase in gut permeability seen in the leakage of fluorescein isothiocyanate-dextran (4kD) when compared to the blood FICT fluorescence 4 h after lavage in SCI-mice 7 days post-SCI. They further showed that the gut-associated lymphoid tissue (GALT) in mice was activated. Kigerl et al. used VSL#3 probiotic in mice, comprising *Lactobacillus casei, Lactobacillus plantarum, Lactobacillus acidophilus, Lactobacillus debrueckii* subsp *bulgaricus, Bifidobacterium longum, Bifidobacterium breve, Bifidobacterium infantis* and *Streptococcus salivaris* subsp *thermophiles*, from the day of injury to 35 days post-SCI in mice and showed improved functional recovery, reduced spinal lesion and significant transient gut microbiome enrichment of *Bifidobacteriales* and *Lactobacillales*.

### Spinal cord injury and gastrointestinal tract microbiome in humans

Five case-control studies of persons with chronic traumatic SCI (more than 6 months) and one interventional study with spina bifida compared to healthy able-bodied individuals were included. The studies of Zhang et al. [17, 18] were grouped as they contain the same population. Two studies were assessed with a low risk of bias and four with moderate risks (Supplementary Table 1). Two studies exclusively studied male individuals [17, 18] and two studies lack the description of the extent of injury [19, 20]. One study has a subgroup of acutely injured SCI wherein the individuals had antibiotics for their health management [21] and another included incomplete SCI [19]. All studies targeted 16S rRNA variable regions or microbiome analysis. Summarized findings of the microbiome changes are in Table 2.

**Table 2:** Summary of the findings from the human gut studies.

Study population	Sequencing technique	Most represented bacterial taxa	Microbial differences (vs able-bodied individuals)	Diversity indices (vs able-bodied individuals)	Other significant results	RoB assessment (Author, Publication Year, Country)
Chronic traumatic complete SCI (C-SCI and TL-SCI) vs able-bodied individuals 23 C-SCI, 23 TL-SCI and 20 able-bodied individuals	16S rRNA V4 515F 806R Illumina MiSeq	SCI (g) *Bacteroides, Faecalbacterium, Blautia, Megamonas, Bifidobacterium, Phascolarctobacterium* Cervical SCI (g) *Bacteroides, Faecalbacterium, Blautia, Megamonas, Bifidobacterium, Escherichia-Shigella* Able-bodied individuals (g) *Bacteroides, Faecalbacterium, Megamonas, Prevotella_9, [Eubacterium]_rectale_group*	SCI ↑(g) *Bacteroides, Blautia, Lachnoclostridium, Escherichia-Shigella* ↓(g) *Megamonas, Prevotella_9, [Eubacterium]_rectale_group, Dialister and Subdoligranulum* Cervical SCI ↑ (p) Proteobacteria, Verrucomicrobia (c)Verrucomicrobiae (o) Verrucomicrobiales (f) Bacteroidaceae, Verrucomicrobiaceae (g) *Bacteroides, Akkemansia, Escherichia-Shigella* ↓(p) Firmicutes (f) Prevotellaceae, Ruminoccaceae (g) *Prevotella_9, Faecalbacterium, Megamonas, [Eubacterium]_rectale_group, Dialister*	α diversity Simpson ↓T-SCI Chao1- $\uparrow$ T-SCI, $\uparrow$TL-SCI (vs C-SCI) β diversity (+) significant difference between able-bodied individuals vs C-SCI via UniFrac-PCoA	↑Bifidobacterium in SCI with constipation vs w/o constipation ↑(g) *Megamonas* (SCI bloating) ↑(g) *Alistipes* (SCI w/o bloating) Glucose, HDL and CR had significantly correlated with SCI Serum glucose, HDL, APOA1 and LPA significantly correlated with C-SCI.	Moderate (Zhang et al., 2018^a^ [17, 18], China)
Chronic traumatic complete SCI at T6 and above (UMN vs LMN) and able-bodied individuals 15 UMN, 15 LMN and 10 able-bodied individuals	16S rRNA V4 515F 806R Illumina MiSeq	SCI (g) *Blautia, Bifidobacterium, Ruminococcus, Faecalbacterium, Subdoligranulum* Able-bodied individuals (g) *Faecalbacterium, Blautia, Ruminococcus, Roseburia, Bifidobacterium*	UMN ↓(g) *Pseudobutyrivibrio, Dialister and Megamonas* ↓ (g) *Marvinbryantia* (vs LMN) LMN ↓(g) *Pseudobutyrivibrio, Roseburia and Megamonas*	–	–	Low (Gungor et al., 2016 [22], Turkey)
Chronic traumatic SCI (w/ 22% with complete lesion) vs able-bodied individuals 23 SCI, 23 able-bodied individuals	16S rRNA V3-V4 338F 806R Illumina MiSeq	SCI (g) *Bacteroides, Ruminococcaceae_uncultured, Faecalbacterium, Subdoligranulum,Lachnospiraceae_incertae_sedis* Able-bodied individuals (g) *Bacteroides, Ruminococcaceae_uncultured, Lachnospiraceae_incertae_sedis, Faecalbacterium, Escherichia-Shigella*	↑(g)*Incertae_sedis, Parabacteroides, Allistipes, Phascolarbacterium, Christensenella, Barnesiella, Anaerotruncus, Holdemania, Eggerthella, Intestimonas, Gordonibacter, Bilophila, Flavonifractor, Coprobacillus* ↓(g) *Haemophillus, Clostridium_sensu_Stricto_1, Veillonella, Dialister, Roseburia, Megamonas, Leuconostoc, Lachnospira, Megasphaera, Rhodococcus, Ruminococcus, Subdoligranulum, Pseudobutyrivibrio, Faecalbacterium*	α diversity (−) Shannon (−) Simpson (−) Chao1 (−) ACE (−) Observed OTUs β diversity (+) significant difference between groups by UniFrac-PCoA	–	Moderate (Lin et al., 2020 [19], China)
Complete traumatic SCI (A-SCI and L-SCI) vs able-bodied individuals 7 A-SCI, 25 L-SCI and 20 able-bodied individuals	16S rRNA V3-V4 primers unspecified ASV clustering taxa assignment	–	SCI ↑(f) Erysipelotrichaceaea, Acidaminococcaceae, Rikenellaceae, (g) *Lachnoclostridium, Eisenbergiella, Alistipes, Oscillibacter and Anaerotruncus* L-SCI ↑(o) Clostridiales (f) Lachnospiraceae, Eggerthallaceae ↓(o)Bacillales (g) *Campylobacter* ↓(f) Burkholderiaceae (vs Able-bodied individuals and A-SCI) A-SCI ↑(f) Desulfovibrionaceae, Odoribacter, Marinifiliaceae (g) *Sutterella* (vs control and L-SCI) ↓(g) *Haemophillus, Clostridium*	α diversity (−) Shannon (−) Simpson (−) Phylogenetic diversity ↑Chao1 ↑Species count (−) Shannon (A-SCI vs L-SCI) (−) Simpson (A-SCI vs L-SCI) (−) Phylogenetic diversity (A-SCI vs L-SCI) ↑Chao1 (A-SCI vs L-SCI) ↑Species count (A-SCI vs L-SCI) β diversity (+) significant dissimilarity between 3 groups by Bray–Curtis dissimilarity-PCoA, UniFrac-PCoA except for unweighted UniFrac-PCoA of A-SCI vs L-SCI	–	Low (Li et al., 2020 [21], United States)
Pediatric individuals with spina bifida Spina bifida (16), able-bodied individuals (10) Intervention: transanal irrigation	16S rRNA V1-V2 FOH-27Fmod ROH-338R Illumina MiSeq	SCI (g) *Bacteroides, Faecalbacterium, Blautia, Ruminococcus, Prevotella* Able-bodied individuals (g) *Bacteroides, Prevotella, Bifidobacterium, Ruminococcus, Faecalbacterium*	↓(g) *Faecalbacterium, Oscillospira, Blautia, Roseburia, Lachnospira, Dialister*		Transanal irrigation ↑Bristol score ↓neurogenic bowel dysfunction score –changes in the gut microbiota ↑*Bacteroides* ↑*Roseburia* ↓*Turicibacter* (+) correlation on the relative abundance of *Roseburia* and Bristol score	Moderate (Furuta et al., 2021 [20], Japan)

*RoB* risk of bias, *SCI* spinal cord injury, *UMN* upper motor neuron, *LMN* lower motor neuron, *A-SCI* acute spinal cord injury, *L-SCI* long-term spinal cord injury, *C-SCI* cervical spinal cord injury, *TL-SCI* thoracolumbar spinal cord injury, *PCoA* principal coordinate analysis, *ANOSIM* analysis of similarities, *ASV* amplicon sequencing variant, *s* species, *g* genus, *f* family, *o* order, *p* phylum, *OTU* operational taxonomic units.

^a^Zhang et al. 2019 was lumped with Zhang et al. 2018 as they have same control and C-SCI population.

Five of the studies included adult populations with two studies subdividing their study population by level of SCI. The Zhang et al. [17, 18] studies recruited only male individuals with chronic thoracolumbar SCI (TL-SCI) or cervical-SCI (C-SCI) and compared them to healthy able-bodied individuals. The TL-SCI group had significantly higher Chao1 and lower Simpson index vs able-bodied individuals. There was a significant difference in β diversity at the phylum level across the three groups. Among the top OTUs *Bacteroides and Blautia* were increased in SCI while *Prevotella*, and *[Eubacterium] rectale* showed a significant decrease in SCI and *Faecalbacterium* decreased only in C-SCI [17, 18]. Significant serum level correlations of high-density lipoprotein (HDL) (negative) and apoA1 (positive) with the changes at microbial genus levels. Low-density lipoprotein and glucose positively correlated with changes at phylum level in C-SCI. Additionally, the team noted that the presence of *Bacteroides* and *Blautia* were significantly associated with lipid metabolism in C-SCI than in able-bodied individuals and *Faecalbacterium, Megamonas* and *Prevotella* correlated negatively with lipid metabolism biomarkers while *Lactobacillus* correlated positively to glucose [17]. In contrast, Gungor et al. [22] classified their study based on the bowel dysfunctional manifestation of individuals with SCI and only included individuals with SCI above T6 or cauda equina syndrome. They found that the gut microbiome in SCI was dominated by *Blautia, Bifidobacterium, Faecalbacterium*, and *Ruminococcus* and between groups, *Roseburia, Pseudobutyrivibrio, Dialister, Marvinbryantia*, and *Megamonas* were significantly different. The authors concluded that there was a significant reduction of butyrate-producing bacteria among individuals with SCI.

Lin et al. [19] recruited individuals with chronic traumatic SCI but only 22% had complete SCI. The extent of loss of bowel function loss was not described. They found no significant difference in α diversity but β diversity was significantly different between individuals with SCI vs able-bodied individuals. They showed enrichment of *Parabacteroides, Alistipes, Phascolactobacterium, Eggerthella*, *Intestimonas, Flavonifractor, Christensella, Barnesiella, Holdemama, Gordonibacter, Bilophila*, and *Coprobacillus* while depletion of *Haemophilus, Clostridium sensu stricto1, Veillonella, Dialister, Roseburia, Megamonas, Subdoligranulum, Leuconostoc, Lachnospira, Megasphaera, Rhodococcus, Ruminococcus, Pseudobutyrivibrio*, and *Faecalbacterium* in individuals with SCI. Li et al. [21], on the other hand, compared patients with acute SCI and those with chronic SCI to able-bodied individuals. They observed that the α diversity indices were sensitive to species count and were significantly higher among individuals with SCI and those with acute SCI had significantly higher diversity compared to the chronic SCI group. The significant composition differences observed between the groups were bacterial genera changes that are linked to metabolic and neurologic disorders, antibiotic use, and intestinal inflammation.

Furuta et al. [20] investigated the effect of transanal irrigation in improving constipation among pediatric spina bifida individuals’ vs healthy able-bodied individuals. Transanal irrigation significantly improved constipation symptoms as measured with Bristol scores and neurogenic bowel dysfunction scores. *Faecalbacterium, Oscillospira, Blautia, Roseburia, Lachnospira, Dialister* were significantly decreased among individuals with spina bifida and *Roseburia* correlated positively with the Bristol scores. The use of transanal irrigation significantly increased *Bacteroides* and *Ruminococcus*, and decreased *Turicibacter*.

### Spinal cord injury and urinary tract microbiome

There were six studies on the urinary tract microbiome. Five studies were on adult populations with neurogenic bladder dysfunction secondary to SCI. One study reported a subgroup of children with spina bifida [23] and one was exclusively on children [24]. All studies utilized varying DNA extraction methods but all used the 16S rRNA variable regions for sequencing. All studies showed polymicrobial urine in individuals with SCI. Summarized findings of the microbiome changes are shown in Table 3. All the urine studies had moderate risk of bias. The studies did not account for confounding variables and the extent of injury in the study populations were not sufficiently described [23–28] (Supplementary Table 1).

**Table 3:** Summary of the findings from the human urinary tract studies.

Study population (*N*) and intervention	DNA extraction	Sequencing technique	Most represented bacterial taxa in SCI (%)	Microbial differences in SCI vs able-bodied individuals	Diversity indices	Other significant results	RoB assessment (Author, Publication Year, Country)
Adults with SCI (5) and children with spina bifida (5) Intervention: intravesical installation of *Lactobacillus rhamnosus*	DNeasy Kit and QIAmp DNA Micro Kit (Qiagen)	16S rRNA Primers unspecified Illumina MiSeq	(g) *Escherichia* (20.7), *Streptococcus* (16.4), *Prevotella* (12.8), *Pseudomonas* (10.5) *Veillonella* (7.3), *Lactobacillus* (6)	–	α diversity (−) Chao1 (−) Shannon (−) Phylogenetic diversity β diversity —significant difference between adults and children via Unifrac-PCoA	*Escherichia, Prevotella, Lactobacillus, Streptocococcus* and *Veillonella* significantly changed in proportions before and after instillation	Moderate (Forster et al., 2019 [23], United States)
Population: adult males with SCI (3) Intervention: probiotic (*Lactobacillus rhamnosus* with *Lactobacillus.reuteri* and *Lactobacillus rhamnosus* with *Bifidobacterium)*.	FastDNA SPIN Kit for Soil and the FastPrep Instrument (MP Biomedicals, Santa Ana, CA)	16S rRNA 27F/536R Illumina MiSeq Grengenes	(f) Enterobacteriaceae (48), *Providencia* (11), unclassified genus of Alcaligenaceae (10), unclassified genus of Enterococcaceae (9), *Pseudomonas* (4), *Veillonella* (4)	–	α diversity —significantly different Shannon diversity index for the 3 individuals β diversity —significant difference across the 3 individuals by UniFrac-by nonmetric multidimensional scaling	Changes caused by probiotic use or infection were transient and goes back to pre-treatment communities	Moderate (Bossa et al., 2017 [25], Australia)
Population: adults with SCI (3) and spina bifida (3) Intervention: intradetrusor botulinum	Extraction via silica columns	16S rRNA V3-V4 Primers unspecified Illumina	(g) *Escherichia, Klebsiella, Lactobacillus, Enterococcus*	–	–	Intradetrusor botulinum decreased UTI after 6 months observation Urine culture results were confirmed by sequencing	Moderate (Philippova et al., 2020 [26], Russia)
Population: adults with SCI (27) and able-bodied individuals (26) Intervention: none	Phenol-chloroform-isoamyalcohol extraction with enzymatic digestion and phyical lysis for lysate	16S rRNA V1-V3 27F/534R 454 sequencing and aligned with SILVA database	(g) *Lactobacillus, Klebsiella, Corynebacterium, Staphylococcus, Streptococcus, Gardenella, Aerococcus, Prevotella, Escherichia, Brevibacterium, Salmonella, Proteus, Enterococcus, Veillonella, Atopobium*	(g) *Corynebacterium, Staphylococcus, Streptococcus, Peptoniphilus, Lactobacillus, Escherichia, Salmonella, Enterococcus, Aerococcus, Klebsiella, Proteus, Brevibacterium* Significant ↑ (g) *Klebsiella, Escherichia* and Enterococcus in NB	β diversity —Similarity in microbiome composition of able-bodied individuals vs 0–2 mos duration SCI and 13–48 mos vs 48+ mos duration by PCA	(g) *Lactobacillus, Corynebacterium, Gardnella, Prevotella and Enterococcus* define gender difference (o) Enterobacteriales could be an indicator for UTI (g) *Aerococcus* and *Enterococcus* maybe important indicators for catheter use or bacteriuria (g) *Lactobacillus* and *Streptococcus* associates with healthy bladders except *Lactobacillus iners* and *Streptococcus salivarius*	Moderate (Fouts et al., 2012 [28], United States)
Population: adults with SCI (24) and able-bodied individuals (23) Intervention: none	Phenol-chloroform-isoamyalcohol extraction with enzymatic digestion and phyical lysis for lysate	16S rRNA V1-V3 27F/534R 454 sequencing and aligned with SILVA database	–	↑ (g) *Lactobacillus, Gardnella* and *Enterobacter* in females vs males ↑ (s) *Lactobacillus iners* and *Gardnella vaginalis* in females vs males *↑* (s) *Enterococcus faecalis, Pseudomonas aeruginosa, Klebsiella pneumonia, Escherichia coli* *↑* (f) Enterobacteriaceae to those using SP and IC but not those who can void ↓ (f)Lactobacillaceae in NB using to those using SP and IC but not those who can void	α diversity (−) Shannon (−) Chao1 (−) Inverse Simpson (−) Fisher β diversity —no significant difference by gender or presence of pyuria.	(f) Lactobacillaceae and (s) *Lactobacillus crispatus* characterized a female able-bodied individual (f) fStreptococcaceae and (s) *Staphylococcus haemolyticus* characterize a male able-bodied individual (g) *Actinobaculum* was associated with pyuria	Moderate (Groah et al., 2016^a^ [27], United States)
Children with NB Intervention: none		16S rRNA Primers unspecified Illumina MiSeq	(f) Enterobacteriaceae (56), *Klebsiella* (19), *Staphylococcus* (7), *Streptococcus* (3) and non-specified family Neisseriaceae (3) NGC *Staphylococcus* (38), non-specified family Neisseriaceae (21), Enterobacteriaceae (17), *Gemella* (5) AB Enterobacteriaceae (65), *Klebsiella* (17), *Staphylococcus* (5), *Streptococcus* (4) *Enterococcus* (4) UTI Enterobacteriaceae (55), *Klebsiella* (26), *Staphylococcus* (11), *Streptococcus* (5) *Enterococcus* (3)		α diversity (−) Shannon (−) Chao1 β diversity —UniFrac-PCoA showed significant overlap without clustering between groups		Moderate (Forster et al., 2020 [24], United States)

*RoB* risk of bias, *SCI* spinal cord injury, *NGC* no growth in culture, *AB* asymptomatic bacteriuria, *UTI* urinary tract infection, *NB* neurogenic bladder, *PCA* principal component analysis, *PCoA* principal coordinate analysis, *s* species, *g* genus, *f* family, *o* order, *p* phylum, *mos* months.

^a^Groah et al. is a re-analysis of Fouts et al using a different bioinformatics tool.

Three studies had interventions with two of the studies investigating a probiotic treatment. The study of Forster et al. [23] was a phase 1a clinical trial on intravesical instillation of *L. rhamnosus* in children with spina bifida and adults with traumatic SCI. No significant changes in α diversity index between the pre and post-instilation microbiomes were detected, but the microbiome composition between children and adults was significantly different. They observed that the majority of bacteria pre-instillation and post-instillation were the same but significantly changed in proportions. On the other hand, Bossa et al. [25] recruited by convenience three individuals from the ProSCIUTTU clinical trial on probiotics containing *L. rhamnosus* with *L. reuteri* and *L. rhamnosus* with *Bifidobacterium* and observed for their urine catheter flora. They were observed longitudinally for 6 months during the probiotic treatment and for another 2 years thereafter. Two of the patients had no clinically significant UTI while one had a single episode requiring catheter change during the study. Analysis of the terminal restriction fragment polymorphism and the sequences between patients was significantly changed. Among patients, the microbiome was similar pre and post-treatment with probiotics. Their longitudinal analysis showed that the microbiome changed with the onset of probiotics or the presence of symptomatic UTI but reverts to baseline microbiome profile. Philippova et al. [26] studied the effect of intradetrusor botulinum in 6 adults (2 males, 4 females) with SCI from trauma or spina bifida. The intervention decreased the occurrence of symptomatic UTI after 6 months of observation. All the urine microbiome prior to intervention was dominated by Enterobacterales but three of the females were shifted to predominantly *Lactobacillus* urine microbiome. The urine culture result was confirmed by the sequencing data with *Escherichia, Klebsiella, Lactobacillus*, and *Enterococcus* as the most common bacterial genera identified.

Fouts et al. [28] included healthy abled body individuals and compared to individuals with SCI who voided spontaneously (30%), with intermittent catheterization (30%), and with indwelling catheters (40%); females represented 44% of the population. They found that the healthy able-bodied individuals’ urine microbiome was similar up to 2 months after -SCI but was different in those with 13 months and more after SCI. The presence of *Enterococcus* and *Escherichia* in chronic cases significantly contributed to the differences observed. Groah et al. [27] re-analyzed the data using a newer bioinformatics tool and found *Escherichia coli* and *Enterococcus faecalis* were significantly enriched along with *Pseudomocas aeruginosa* and *Klebsiella pneumoniae* in SCI. They likewise showed that *Lactobacillus crispatus* was significantly more abundant in female able-bodied individuals vs females with SCI and *Staphylococcus haemolyticus* in male able-bodied individuals vs males with SCI. Moreover, they found that members of Enterobacteriaceae were significantly enriched while Lactobacillaceae were significantly depleted among those persons with SCI using suprapubic catheters and intermittent catheterization compared to able-bodied individuals but not to those persons with SCI who could void. The diversity indices across all groups and sex, presence of pyuria was not significantly different. There were on average 18 phylotypes (taxon-neutral phylogenic types) that supported the idea of polymicrobial urine.

Forster et al. [24] studied children with neurogenic bladder due to myelomeningocele, anorectal malformation, and tethered cord and who were using intermittent catheterization. They found no significant difference in the α diversity indices and no significant difference in β diversity. Members of Enterobacteriaceae was the predominant bacteria identified in those with asymptomatic bacteriuria and UTI while *Staphylococcus* was the dominant genera among those with negative urine culture after sequencing.

## Discussion

### Gut microbiome of individuals with SCI

Studies on gut microbiomes among individuals with SCI are limited. They indicate that there is gut dysbiosis post-SCI, verified by changes in the microbiome analysis in both animal studies and human clinical studies. There is a consistent significant β diversity difference in gut microbiome composition between individuals with SCI and able-bodied individuals. The α diversity indices are inconsistent. SCI seems to induce gut leakiness and bacterial translocation leading to an imbalance in immune response resulting in persistent inflammation. The dominant bacterial genera remain unchanged post-SCI but butyrate-producing bacteria such as *Faecalbacterium, Megamonas, Roseburia* are significantly depleted and inflammation-associated bacteria like *Alistipes, Anaerotruncus*, and *Lachnoclostridium* are enriched. Interventions such as probiotics, melatonin, and FMT could reverse some of the clinical effects and the dysbiosis associated with SCI.

The gut microbiome of individuals with SCI is significantly changed with the loss of autonomic nervous system innervation. The loss of control of gut functions and the observed increase in gut permeability allows faster movement of metabolites and gut microbes to cross to blood circulation and induce systemic inflammation [16]. These events change the way the immune system interacts with the gut microorganisms and how they are regulated [1, 3], leading to dysbiosis with a consistent decrease of major butyrate-producing bacteria. Butyrate, a short-chain fatty acid produced by fermentation in the colon, is an energy source for epithelial cells and able to stimulate the production of mucin, antimicrobial peptides, and tight junction proteins which leads to an improved gut mucosal barrier, modulation of the immune system regulation regarding gut microorganisms and reduces oxidative stress in the colon [29]. Similar to SCI, in clinical and preclinical studies of diseases with chronic inflammation such as inflammatory bowel diseases [30, 31], type 2 diabetes [32, 33], and atopic dermatitis [34], gut dysbiosis is characterized by depletion of butyrate-producing bacteria. Moreover, enriched bacterial genera such as *Alistipes, Anaerotruncus*, and *Lachnoclostridium* are associated with inflammation and obesity [35–41]. Individuals with SCI are at risk of developing obesity and metabolic disorders [42]. Increasing adiposity among this population is both a consequence of their limited physical activity resulting in positive energy balance but results as well in systemic inflammation associated with chronic SCI [43]. The gut dysbiosis observed has been likewise linked to anxiety, mood, behavior symptoms, and susceptibility to infection [1, 3, 4].

The α diversity of the gut microbiome did not significantly differ consistently in SCI compared to able-bodied individuals. After SCI a new gut environment could allow new species to proliferate for some time but eventually, these are outgrown by predominant species resulting in a lack of change in species abundance distribution. These observations are time-limited. The more acute the observation, the more α diversity could change significantly. The time of measurement in the animal studies in the review ranged from 3 to 56 days post-SCI but the clinical studies all included individuals with chronic SCI except for one. This lack of similar time observation could account for the inconsistency observed.

### Urinary tract microbiome of individuals with SCI

There are very few studies on the urinary tract microbiome but the results establish that urine among individuals with SCI is polymicrobial. Members of Enterobacteriaceae (*E. coli, K. pneumonia*) predominantly populate the urine in SCI in the review. The use of probiotics and antibiotics induce transient but significant shifts in the urinary tract microbiome. The microbial composition of urine microbiome of individuals with SCI vs able-bodied individuals is not significantly different regardless of sex. The microbial composition among children’s urine is similar in those with UTI, asymptomatic bacteriuria, or negative culture, but significantly differs from the composition in adults.

Urine in individuals with SCI is polymicrobial and similar to published data on able-bodied individuals there is a similarity in the identified bacterial genera but differs in the dominant group. *Lactobacillus* and *Streptococcus* are well represented in able-bodied individuals [44] while members of Enterobacteriaceae such as *E. coli* and *K. pneumonia*e are the dominant bacterial species in the urine of individuals with SCI especially among those already with UTI and asymptomatic bacteriuria. The dominance of pathogenic members of Enterobacteriaceae could be contributory to the increased risk to UTI, recurrent UTI, and development of chronic kidney failure. Symptomatic UTI and recurrent UTI in this population are frequent and caused mostly by *E. coli*, *K. pneumoniae, P. aeruginosa, E. faecalis, Staphylococcus aureus*, and *Proteus mirabilis* [45]. The difference in the microbiome, however, cannot be fully ascribed to SCI especially the increase in Enterobacteriaceae is seen mostly among those who use suprapubic catheters and intermittent catheters but not among those who can void when compared to able-bodied individuals [27]. This raises the possibility that catheter use influences the urinary microbiome as well in the SCI population. Moreover, studies in this review show that there is similar composition of bacterial genera among those with no UTI and those with UTI. There is simply a change in the dominant bacteria when there is UTI. This could explain the observed effect of probiotics and antibiotics which lead to significant but transient changes in the urinary microbiome but the microbiome revert to pre-existing composition after use [25].

### Strengths and limitations of current study and directions for future research

The review summarized only studies with genomic sequencing microbiome results. The use of the collective genome allowed the studies to identify organisms previously unidentifiable and even nonviable organisms in culture. This provides better resolution on which bacteria are present in the body sites. The method has limitations especially regarding collection methods, sequencing technique and bioinformatics tool use could give different results. Low biomass samples such as from urine and skin are prone to contamination and host DNA interference. Different sequencing technique has different DNA quality needs, some process only a specific DNA length, introduce bias when amplification is use and has different error rates for reading nucleotide bases. Bioinformatics tools, likewise, use different reference databases and some have only up to a limited level of taxonomic classification.

The search strategy for the review was broad to accommodate terms for multiple body sites and tried to capture early studies as the indexing term microbiota and microbiome are recent. The studies in the review are mostly observational and provide snapshots of a dynamic process in the gut and the urinary tract. They provide limited information and do not capture the dynamic interactions of the organisms. Likewise, microbiomes are affected by age, sex, diet, physical activity, comorbidities, level of injury, duration of injury, and use of therapeutics [1]. Therefore, future studies would benefit from controlling for more variables directly affecting the microbiome, varying sample locations, and additional time points for observation. Improvement of the sequencing technologies and reduced costs for whole genome sequencing will allow the microbial characterization at the species level. This is important for future studies wherein a single species might drive the change at the genus level or higher taxa. Studies should focus on microbiomes of organ-systems commonly affected by the loss of innervation after SCI particularly the urogenital, gut, and skin that may be contributory to the acute and chronic morbidities seen in this group. Clinical studies on immunology and inflammation are needed in persons with SCI to test whether immune defenses react to changes in the microbiome and why a large number of pathogens especially in the urinary tract are tolerated, as seen in the review.

## Conclusion

Studies on microbiomes in SCI remain limited to the gut and the urinary tract. Dysbiosis post-SCI is present and the microbiome profiles follow patterns associated with diseases with chronic inflammation and metabolic disorders. The urinary tract, on the other hand, shows a microbiome that shifts in composition post-SCI with the dominance of UTI-associated organisms. Dynamic changes in the microbiome can be used clinically as a specific target for therapeutics to correct dysbiosis or to reduce the risk of disease.

## Supplementary information

Supplement A

Supplemental Table 1

## Data Availability

All data generated or analyzed during the current study are included in this published article and its Supplementary information files.
