# Supplementary material for: Systematic review of the changes in the microbiome following spinal cord injury: animal and human evidence
Source: Spinal Cord. 2022 Jan 6;60(4):288–300. doi: 10.1038/s41393-021-00737-y (PMC8989678; doi:10.1038/s41393-021-00737-y)
Supplement: Supplementary file 1 — Supplement A [file 41393_2021_737_MOESM1_ESM.docx]

**Supplement A.** Detailed search strategies used in this review per database

Apr 7th 2021

|  | Before deduplication | After deduplication |
| --- | --- | --- |
| Embase | 5216 | 5190 |
| Medline Ovid | 3649 | 1189 |
| Cochrane CENTRAL | 174 trials | 89 |
| Web of Science | 1090 | 315 |
| Google Scholar | 200 | 86 |
| Total | 10329 | 6869 |

3460 duplicate records have been removed

**Embase.com**

('spinal cord injury'/exp OR 'cervical spine injury'/de OR 'spinal cord ischemia'/de OR 'paraplegia'/de OR 'spastic paraplegia'/de OR 'quadriplegia'/de OR 'spinal dysraphism'/de OR 'neurogenic bowel'/de OR ('injury'/exp AND 'spinal cord'/exp) OR (((spine OR spinal) NEAR/3 (injur* OR trauma* OR damag*)) OR ('spinal cord' NEAR/3 (disease* OR disorder* OR contusion* OR laceration* OR transection* OR lesion* OR trauma* OR post-trauma* OR ischemi* OR ischaemi*)) OR (myelopath* NEAR/3 (trauma* OR post-trauma* OR posttrauma*)) OR ((spine OR spinal OR vertebra*) NEAR/3 (fracture* OR trauma* OR injur* OR damage* OR wound*)) OR 'central cord injury syndrome*' OR 'central cord syndrome*' OR 'central spinal cord syndrome*' OR 'cauda equine syndrome*' OR 'anterior cord syndrome*' OR 'conus medullaris syndrome*' OR 'Brown Sequard' OR paraplegi* OR quadriplegi* OR tetraplegi* OR wheelchair* OR paralympi* OR para-athlet* OR parathlet* OR para-sport* OR ((neurogenic OR neuropathic) NEAR/2 (bowel* OR bladder*)) OR (('lower limb*' OR 'lower extremit*') NEAR/3 paralys*)):ab,ti) AND ('microflora'/exp OR 'microbial consortium'/de OR 'microbial diversity'/de OR 'dysbiosis'/de OR 'bacterial infection'/exp OR 'bacterial load'/de OR 'bacteriuria'/exp OR 'pyuria'/de OR 'Bacteroidaceae'/exp OR (microbi* OR dysbios* OR dys-symbios* OR dysbacterios* OR disbacteri* OR flora OR microflor* OR pyuria* OR bacteri* OR (infect* NEAR/3 (dermal OR cutaneous OR skin))):ab,ti) NOT ([Conference Abstract]/lim OR [Letter]/lim OR [Note]/lim OR [Editorial]/lim)

**MEDLINE (Ovid)**

((exp Spinal Cord Injuries/ or exp Spinal Cord Ischemia/ or exp Paraplegia/ or Quadriplegia/ or Spinal Dysraphism/ or Neurogenic Bowel/ or Neurogenic Urinary Bladder/ or (((spine or spinal) adj3 (injur* or trauma* or damag*)) or (spinal cord adj3 (disease* or disorder* or contusion* or laceration* or transection* or lesion* or trauma* or post-trauma* or ischemi* or ischaemi*)) or (myelopath* adj3 (trauma* or post-trauma* or posttrauma* or injur*)) or ((spine or spinal or vertebra*) adj3 (fracture* or trauma* or injur* or damage* or wound* or lesion*)) or central cord injury syndrome* or central cord syndrome* or central spinal cord syndrome* or cauda equine syndrome* or anterior cord syndrome* or conus medullaris syndrome* or Brown Sequard or paraplegi* or quadriplegi* or tetraplegi* or wheelchair* or paralympi* or para-athlet* or parathlet* or para-sport* or ((neurogenic or neuropathic) adj2 (bowel* or bladder*)) or ((lower limb* or lower extremit*) adj3 paralys*)).ab,ti.) and (microbiota/ or gastrointestinal microbiome/ or microbial consortia/ or Dysbiosis/ or exp Bacterial Infections/ or Bacterial Load/ or Bacteriuria/ or Pyuria/ or (microbi* or dysbios* or dys-symbios* or dysbacterios* or disbacteri* or flora or microflor* OR pyuria* or bacteri* or ((infect*) adj3 (dermal or cutaneous or skin))).ab,ti.)) not (letter or news or comment or editorial or congresses or abstracts).pt

**Cochrane CENTRAL**

((((spine or spinal) NEAR/3 (injur* or trauma* or damag*)) OR ("spinal cord" NEAR/3 (disease* or disorder* or contusion* or laceration* or transection* or lesion* or trauma* or post-trauma* or ischemi* or ischaemi*)) OR (myelopath* NEAR/3 (trauma* or post-trauma* or posttrauma*)) OR ((spine or spinal or vertebra*) NEAR/3 (fracture* or trauma* or injur* or damage* or wound*)) OR "central cord injury syndrome" OR "central cord syndrome" OR "central spinal cord syndrome" OR "cauda equine syndrome" OR "anterior cord syndrome" OR "conus medullaris syndrome" OR "Brown Sequard" OR paraplegi* OR quadriplegi* OR tetraplegi* or wheelchair* or paralympi* OR para-athlet* OR parathlet* OR para-sport* or ((neurogenic OR neuropathic) NEAR/2 (bowel* OR bladder*)) or ((lower NEXT limb* OR lower NEXT extremit*) NEAR/3 paralys*)):ab,ti) AND ((microbi* OR dysbios* OR dys-symbios* OR dysbacterios* OR disbacteri* OR flora OR microflor* OR pyuria* OR bacteri* OR (infect* NEAR/3 (dermal or cutaneous or skin))):ab,ti)

**Web of Science (Core Collection)**

TS=(((((spine or spinal) NEAR/3 (injur* or trauma* or damag*)) OR ("spinal cord" NEAR/3 (disease* or disorder* or contusion* or laceration* or transection* or lesion* or trauma* or post-trauma* or ischemi* or ischaemi*)) OR (myelopath* NEAR/3 (trauma* or post-trauma* or posttrauma*)) OR ((spine or spinal or vertebra*) NEAR/3 (fracture* or trauma* or injur* or damage* or wound*)) OR "central cord injury syndrome" OR "central cord syndrome" OR "central spinal cord syndrome" OR "cauda equine syndrome" OR "anterior cord syndrome" OR "conus medullaris syndrome" OR "Brown Sequard" OR paraplegi* OR quadriplegi* OR tetraplegi* or wheelchair* or paralympi* OR para-athlet* OR parathlet* OR para-sport* or ((neurogenic OR neuropathic) NEAR/2 (bowel* OR bladder*)) or (("lower limb*" OR "lower extremit*") NEAR/2 paralys*))) AND ((microbi* OR dysbios* OR dys-symbios* OR dysbacterios* OR disbacteri* OR flora OR microflor* OR pyuria* OR bacteri* OR (infect* NEAR/3 (dermal or cutaneous or skin))))) AND DT=(article)

**Google Scholar first (most relevant) 200 results (out of 62’000)**

"Spinal cord injury"|"Brown Sequard"|paraplegia|quadriplegia|tetraplegia|wheelchair|paralympic|"neurogenic bowel|bladder"|"lower limb|extremity paralysis" microbiome|dysbiosis|dysbacteriosis|flora|microflora|pyuria|bacterial|"skin|dermal infection
