# Supplementary material for: Systematic review of the changes in the microbiome following spinal cord injury: animal and human evidence
Source: Spinal Cord. 2022 Jan 6;60(4):288–300. doi: 10.1038/s41393-021-00737-y (PMC8989678; doi:10.1038/s41393-021-00737-y)
Supplement: Supplementary file 2 — Supplemental Table 1 [file 41393_2021_737_MOESM2_ESM.docx]

**Supplemental Table 1.** Study quality assessment based on the OHAT Risk Assessment

| Lead Author, Year | Organ System | Selection Bias | | | Confounding Bias | Performance Bias | | Attrition/ Exclusion Bias | Detection Bias | | Risk of BiasTier |
| --- | --- | --- | --- | --- | --- | --- | --- | --- | --- | --- | --- |
|  |  | *Was administered dose or exposure level adequately randomized?* | *Was allocation to study groups adequately concealed?* | *Did selection of study participants result in appropriate comparison group?* | *Did the study design or analysis account for important confounding and modifying variables* | *Were experimental conditions identical across study groups?* | *Were the research personnel and human subjects blinded to the study group during the study?* | *Were outcome data complete without attrition or exclusion from analysis?* | *Can we be confident in the exposure characterization?* | *Can we be confident in the outcome assessment?* |  |
| Schimdt, 2020 | Gut-Animal | ***** | ***** | NA | NA | ****** | ****** | ****** | ****** | ****** | **Low** |
| Schimdt, 2021 | Gut-Animal | ***** | ***** | NA | NA | ****** | ****** | ****** | ****** | ****** | **Low** |
| Jing, 2018 | Gut-Animal | ***** | ***** | NA | NA | ****** | **-** | ****** | ****** | ****** | **Low** |
| O`Connor, 2018 | Gut-Animal | ***** | **-** | NA | NA | ****** | ****** | ****** | ****** | **-** | **Moderate** |
| Myers, 2018 | Gut-Animal | ***** | **-** | NA | NA | ****** | **-** | ****** | ****** | ****** | **Low** |
| Kigerl, 2016 | Gut-Animal | ***** | ***** | NA | NA | ****** | ****** | ****** | ****** | ****** | **Low** |
| Jing, 2021 | Gut-Animal | ***** | ***** | NA | NA | ****** | **-** | ****** | ****** | **-** | **Moderate** |
| Lin, 2020 | Gut-Human | NA | NA | ***** | ***** | NA | NA | ****** | **-** | ****** | **Moderate** |
| Li, 2020 | Gut-Human | NA | NA | ***** | ****** | NA | NA | ****** | ****** | ****** | **Low** |
| Zhang, 2019 | Gut-Human | NA | NA | ***** | **-** | NA | NA | ****** | ****** | ****** | **Moderate** |
| Zhang, 2018 | Gut-Human | NA | NA | ***** | **-** | NA | NA | ****** | ****** | ****** | **Moderate** |
| Gungor, 2015 | Gut-Human | NA | NA | ***** | ****** | NA | NA | ****** | ****** | ****** | **Low** |
| Furuta, 2021 | Gut-Human | NA | NA | ***** | ****** | NA | NA | ****** | **-** | ****** | **Moderate** |
| Forster, 2020 | Urinary Tract-Human | NA | NA | ***** | **-** | NA | NA | ****** | **-** | ****** | **Moderate** |
| Forster, 2019 | Urinary Tract-Human | NA | NA | ***** | **-** | NA | NA | ****** | **-** | ****** | **Moderate** |
| Bossa, 2017 | Urinary Tract-Human | NA | NA | **-** | **-** | NA | NA | ****** | **-** | ****** | **Moderate** |
| Philippova, 2020 | Urinary Tract-Human | NA | NA | **-** | **-** | NA | NA | ****** | **-** | ****** | **Moderate** |
| Groah, 2016 | Urinary Tract-Human | NA | NA | ***** | **-** | NA | NA | ****** | **-** | ****** | **Moderate** |
| Fouts, 2012 | Urinary Tract-Human | NA | NA | ***** | **-** | NA | NA | ****** | **-** | ****** | **Moderate** |

** Definitely Low; * Probably Low; - Probably High/Insufficient Information; -- Definitely High; NA, not applicable
